# Validation of a tele-robotic ultrasound system for abdomen and thyroid gland explorations: a comparison with standard ultrasound

**DOI:** 10.1186/s13089-025-00408-6

**Published:** 2025-01-13

**Authors:** Andreu Antolin, Nuria Roson, Marina Planes, Mar Castillo, Anna Alberti, Manuel Escobar

**Affiliations:** 1 Department of Radiology, Institut de Diagnòstic per la Imatge (IDI), Hospital Universitari Vall d’Hebron, Passeig de la Vall d’Hebron, 119-129, 08035 Barcelona, Spain; 2 Department of Radiology, Hospital Universitari Vall d’Hebron, Passeig de la Vall d’Hebron, 119-129, 08035 Barcelona, Spain

**Keywords:** Tele-robotic ultrasound, Thyroid gland ultrasound, Abdominal ultrasound, Diagnostic ultrasound, Learning curve

## Abstract

**Background:**

Tele-robotic ultrasound (US) is a novel technique that might help overcome the current shortage of radiologists and poor access to radiologists and/or sonographers in remote or rural areas. Despite the promising results of this technology in the past two decades, there is still insufficient data about its advantages and limits, as well as the implementation in routine clinical practice and the learning curve for the user. The purpose of this prospective cohort-based study is to evaluate the performance of a 5G-based tele-robotic US system for abdominal and thyroid gland assessment in a cohort of healthy volunteers and outpatients, as well as assessing the learning curve and patient satisfaction.

**Results:**

64 participants (23 male, 41 female) were consecutively included during the recruitment period, for a total of 51 abdominal and 37 thyroid gland US studies. The mean age was 45.23 ± 18.90 years old, and the body mass index of the abdominal cohort was 22.97 ± 2.95 kg/m^2^. The learning curve estimated a minimum of 20 patients for abdominal tele-robotic US training, being almost non-existent in the thyroid gland cohort. All the variables showed no-statistical differences between standard US and tele-robotic US in the abdominal post-trained cohort except the visualization of the left kidney short axis and its interpolar length. Thyroid gland variables showed no statistical differences. The mean time of exploration for the tele-robotic US for abdomen and thyroid gland examinations were 18.33 ± 6.26 min and 4.64 ± 0.97 min respectively. Most participants (> 70%) felt comfortable and safe while being examined by the tele-robotic US.

**Conclusion:**

Tele-robotic US achieves equal performance in comparison with the standard US when evaluating abdominal structures in this cohort of patients, as well as a relatively fast learning curve and good patient satisfaction. The performance when assessing the thyroid gland is almost identical to the standard US, which makes it a strong first candidate for a future clinical implementation.

**Supplementary Information:**

The online version contains supplementary material available at 10.1186/s13089-025-00408-6.

## Background

There is a global shortage of radiologists due to the imbalance between the number of trained radiologists and the increasing demand of medical imaging in current health practice, with an imperative need for short-term solutions [1]. This problem is aggravated in primary care and rural or remote communities that do not have enough access to a radiologist or sonographer to perform ultrasound (US) examinations, which require physical presence.

Tele-medicine has been consolidated as a valuable tool to overcome some of these drawbacks to better allocate medical resources and access to healthcare, widely spread in the radiology community to remotely report computed tomography and magnetic resonance imaging studies [2, 3].

Tele-robotic US is a novel technology that allows radiologists and sonographers to perform US examinations from a distance, which can help overcome the geographic restrictions that some remote and rural communities have [4]. The first models appeared in the late 90’s and beginning of twenty-first century, and in the past two decades some models have been commercialized for clinical use [5]. However, there is still insufficient data to fully adopt this new technology, especially the learning curve for the radiologist or sonographer.

The aim of this prospective study is to evaluate the performance of a 5G-based tele-robotic US system for abdominal and thyroid gland assessment in a cohort of healthy volunteers and outpatients and to investigate the learning curve of such technique as well as patient satisfaction.

## Materials and methods

### Study design

A prospective unicentric comparative cohort-based study was designed for assessing the reliability of tele-robotic US following the Standards for the Reporting of Studies of Diagnostic Accuracy (STARD) [6]. A total of 64 participants were consecutively included from January 2023 to October 2023. The inclusion criteria were: ≥ 18 years old and healthy volunteers or outpatients without relevant diseases or clinical background. Patients with major diseases such as advanced chronic hepatitis/cirrhosis and cardiopulmonary or neurologic diseases that cause mobility impairment or breathing difficulty were excluded, as well as any other clinical condition that restricts visualization and/or collaboration when performing the US. Outpatients without relevant clinical history and minor medical US requests (such as ruling out cholelithiasis or urolithiasis) were eligible. Participants were recruited during regular shifts. A description of the aim of the study and the methodology was verbally explained to all the eligible participants and were consequently included in the study once the informed consent was signed. The recruitment and US studies were simultaneously done in Hospital Vall Hebron, a university hospital in Barcelona, Spain.

For every included participant a two-step process was designed. First, a single abdominal radiologist (radiologist A) with approximately five years of experience in US performed a tele-robotic US of the thyroid gland or abdomen, or both. Posteriorly, in a double-blind manner, a second independent radiologist with experience in abdominal and thyroid US performed the respective standard US. A total of three abdominal radiologists (B, C and D) with different experiences performed the standard US. Radiologist B, with approximately 20 years of experience in US, did most of the standard studies because of better availability. Radiologist C and D had nine years and six years of experience respectively. In outpatients only the requested US was done, either abdominal or thyroid gland. Healthy volunteers were subject to both US unless specified otherwise by the participant. In the latter, a new US request was made in the electronic clinical record of the patient and any relevant finding reported in the standard US was communicated for further assessment.

At the end of the process, an electronic survey was given to all participants to evaluate the satisfaction and share opinions between both techniques, discriminating between thyroid and abdominal studies.

### Data collected

For all the participants demographic variables were recorded independently of the US performed: age (years), sex (male/female) and body mass index (BMI, Kg/m^2^). The medical request for the US in outpatients was also recorded, as well as incidental findings while performing both techniques. Thyroid nodules were described and classified based on the Thyroid Imaging, Reporting and Data System (TI-RADS) classification [7].

A set of qualitative and quantitative variables were designed to compare both techniques, detailed in table S1 and table S2. Qualitative variables were assessed in all the participants. Quantitative variables were assessed in the last 31 participants since it was when radiologist A was comfortable with managing the robotic arm and hence following the protocol. Left and right hepatic lobe and long/short axis of right and left kidney were also used to establish a retrospective cut-off value (minimum number of explorations) to have proper training in abdominal tele-robotic US.

Finally, the time needed for the radiologist to perform the tele-robotic US was also registered for every case. It was defined as the time from the start of the robotic arm movement until it got back to resting position.

The items evaluated in the participant survey are graded in a 5-point Likert scale and are as follows: (1) I felt comfortable when being examined by the tele-robotic US; (2) I felt safe when being examined by the tele-robotic US; (3) The communication with the radiologist was fluent when being examined by the tele-robotic US; (4) I got my questions about the procedure answered before being examined by the tele-robotic US; (5) I trust the results obtained with the tele-robotic US; (6) The tele-robotic US is more uncomfortable than the standard US; (7) I felt a higher pressure in the examined zone with the tele-robotic US than with the standard US; (8) I prefer the tele-robotic US rather than the standard US; (9) I would be comfortable with the implementation of the tele-robotic US in the future.

### Tele-robotic US protocol

#### Abdominal protocol

The abdominal protocol requires the patient in supine position with the arms behind the head. The examination begins with the transducer placed in sagittal orientation in the epigastric region to assess the left hepatic lobe in different planes. Afterwards it follows the anterior subcostal plane to continue the examination of the rest of the liver and related structures. Then the patient is asked to tilt a little bit towards the left to access the intercostal spaces, and then is positioned in almost complete left lateral decubitus to be able to examine the right kidney. Posteriorly, the patient is asked to move to a supine position to examine the pancreatic region in the epigastrium. To continue with the left hypochondrium structures and left kidney the patient is asked again to move to an incomplete or complete right lateral decubitus to assess the spleen and left kidney. Finally, the patient returns to the standard supine position to evaluate the retroperitoneal main vessels and afterwards the urine bladder in axial and sagittal plane in the hypogastric region. The patient's position is slightly modified in the different steps described based on the morphological characteristics and is asked to help with breathing to better depict the evaluated structures.

#### Thyroid gland protocol

The protocol used for the assessment of thyroid gland requires the patient to be in supine position with neck hyperextension. The transducer is placed in axial orientation in the infrahyoid neck, in the midline, for a full view of the thyroid gland and the isthmus. The transducer is then displaced to right and left to assess both thyroid lobes in short and long axis. Afterwards, the main cervical vessels from both sides are assessed moving the transducer a little bit more laterally. Finally, the right and left submandibular glands are evaluated in the submandibular space with the patient doing a head tilt to the left and right respectively.

An assistant was present in each tele-robotic US examination to assist in the procedure. The role of the assistant is to welcome the patient and ensure that the study is performed without any inconvenience. The assistant accommodates the patient and has direct communication with the radiologist to apply sonographic gel, relocate the robotic arm or camera, assist with the patient’s movements or solve any other potential issues. Patient-site assistants had no prior training in US but were instructed in basic operations of using the system, including turning on and off each component of the system and using the emergency button for shutting down the robotic arm.

The standard US protocol was similar to the previously described with variations depending on the radiologists and the specific clinical request in outpatients.

### Technical characteristics

The MGIUS-R3 model from MGI Tech Co., Ltd. (Shenzhen, China) was used for performing the tele-assisted robotic US. It consists of two parts named doctor’s end and patient end, which are connected via a fast, low latency and large bandwidth 5G network with < 200 ms of delay. In this study both stations were in separate rooms inside the hospital’s Radiology Department.

The doctor’s end is from where the radiologist performs the remote US. It is a station that includes the robot-control console, US control panel with real-time image display and an audiovisual communication system with the patient’s side. The robot-control console allows the remote movement of the robotic arm via a mock US probe with six degrees of freedom (DOF): three degrees for rotation, two for two-dimensional plane, and one for the force sensor, and a simulation panel. An ergonomic elbow rest is located next to the simulation panel to relieve arm fatigue. The audiovisual communication system allows direct visualization of the remote robotic arm to help maneuvering it. It also has direct communication with the patient and the assistant for support (such as holding breath or changing position to facilitate the exam). The parameters of the US such as frequency, gain, parameter measurement, etc., can be adjusted in real time thanks to the US control panel, with real-time display of the US images [8–10].

The patient’s end is a portable device consisting of an US system, a robotic arm and an audiovisual communication system. The robotic arm is designed to accommodate two US probes: abdominal probe (C5-1 with 2.5–5.0 MHz) and a shallow probe (L15-5 with 5.0–12.0 MHz). The robotic arm has a six-dimensional force sensor to obtain real-time feedback and ensure a stable contact force for patient safety. Moreover, the force range of the arm can be manually modified between 3 and 40 N. This dual system contributes to patient safety, and it is also provided with an “emergency stop button” [9, 10]. It is also possible to increase or decrease the probe pressure by exercising or not force with the mock probe into the simulation panel surface. A color bar in the user interface gives a visual representation of the pressure applied, with a minimum and a maximum.

Pictures of the doctor’s and patient’s end and US images for abdominal and thyroid gland explorations are depicted in figures S1-S5.

The standard US was performed with an Aplio i700 model from Canon Medical Systems Corporation (Otawara, Japan) with 1.8–6.2 MHz convex transducer for abdominal US and 3.8–10 MHz lineal transductor for thyroid gland US.

### Ethical issues

The study was approved by the hospital’s Clinical Research Ethics Committee with code PR(IDI)353/2022, and in all cases oral and written informed consent was obtained. Anonymity and data confidentiality (access to records, data encryption and data storage) were guaranteed throughout the research process. Data was prospectively encrypted and collected in a database sheet format that only the principal investigator and collaborators had access to. Confidential patient information was protected in accordance with the European General Data Protection Regulation (GDPR).

### Statistical analysis

Statistical description of qualitative variables was assessed by frequency and percentage. Statistical description of quantitative variables was assessed by mean, standard deviation, and interquartile range.

Statistical analysis for qualitative variables was performed with two-tailed Barnard’s test. It was calculated in the abdominal and thyroid gland cohorts, as well as in the pre-trained and post-trained cohort in the case of the abdominal explorations.

The cut-off value to differentiate between pre-trained and post-trained cohort in the abdominal subgroup was calculated based on Cohen’s kappa coefficient. It was calculated iteratively starting with the full cohort and excluding one extra consecutive observation in each iteration, beginning with the first one. Results were plotted and a cut-off value was subjectively assessed based on the visual representation. Exponential regression was used to approximate the distribution of the plotted data, and an Analysis of Variances (ANOVA) test was used to compare predicted to real values.

Statistical analysis of quantitative variables between two groups with paired data was performed using paired sample Student's t-test for normal variables and Wilcoxon test for non-normal variables.

Statistical significance in all cases was accepted at p < 0.05. All statistical data were evaluated with Python v.3.11 and the libraries scikit-learn v.1.4.0 and SciPy v.1.12.0

## Results

### Study population

**Fig. 1:**
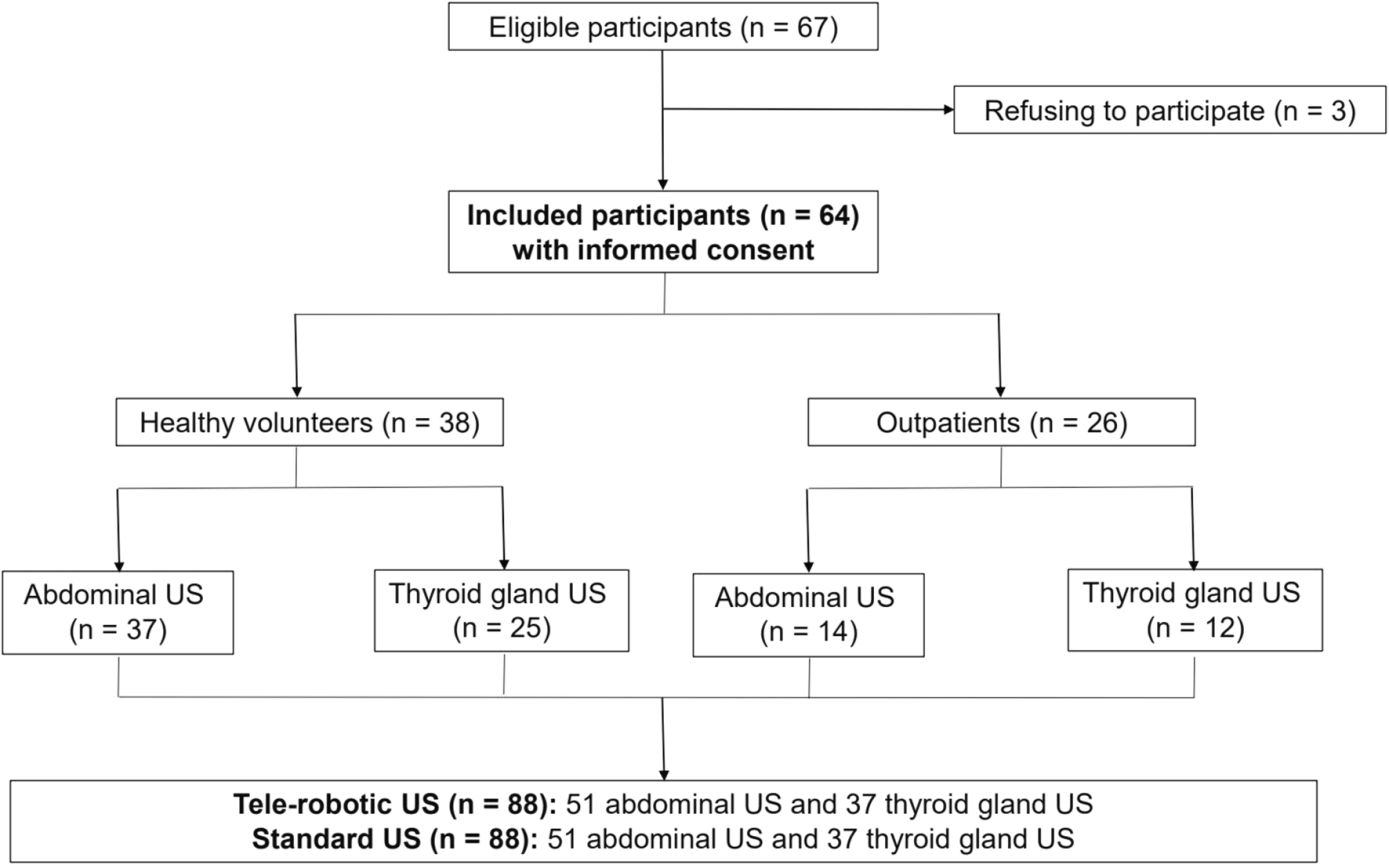
Flow diagram of the participants included in the study divided in healthy volunteers and outpatients, with the total amount of abdominal and thyroid ultrasounds (US)

64 participants were consecutively included during the recruitment period. A total of 176 US were performed during the duration of the study, including 88 tele-robotic US and the correspondent 88 standard US: 51 abdominal and 37 thyroid gland. 24 of the 38 healthy volunteers that participated in the study gave consent for both US, abdominal and thyroid gland, and the remaining 14 only one of them. Figure 1 illustrates the flow of the participants through the study.

The mean age and standard deviation of the 64 participants was 45.23 ± 18.90 years, including 23 male and 41 female. The 51 participants (21 male and 30 female) in the abdominal US subgroup had 41.45 ± 17.78 years, with a mean BMI of 22.97 ± 2.95 kg/m^2^. The 37 participants (13 male and 24 female) in the thyroid US subgroup had 42.95 ± 19.90 years, with a mean BMI of 24.5 ± 4.21 kg/m^2^. Basic demographics and characteristics of the participants are depicted in Table 1. Details about the specific US requests for the outpatients are presented in Table 2.

**Table 1:** Participants’ basic demographics and characteristics

Characteristics	Main Cohort	Thyroid US Cohort	Abdominal US Cohort
Patients (n)	64	37	51
Sex			
Male	23 (35.9)	13 (35.1)	21 (41.2)
Female	41 (64.1)	24 (64.9)	30 (58.8)
Age (years)	45.23 ± 18.90 (32.50)	42.95 ± 19.90 (34.50)	41.45 ± 17.78 (28)
Height (m)	1.69 ± 0.09 (0.11)	1.69 ± 0.09 (0.14)	1.71 ± 0.08 (0.09)
Weight (Kg)	67.44 ± 10.81 (15)	69.30 ± 10.4 (16.5)	67.41 ± 10.28 (14)
Body Mass Index (Kg/m^2^)	23.65 ± 3.95 (4.53)	24.50 ± 4.21 (5.09)	22.97 ± 2.95 (3.27)

Categoric data is expressed as absolute value, with percentage in parentheses

Numeric data is expressed as mean ± standard deviation, with interquartile range in parentheses

**Table 2:** List of the specific clinical requests for abdominal and thyroid gland ultrasounds of the outpatients

Requests for Abdominal Ultrasound
2 patients with cholestasis and right mild hypochondrial pain
1 patient with transaminitis and psoriatic arthritis previously treated with methotrexate
5 patients with transaminitis and no specific clinical symptom
1 patient with clinical suspicion of Gilbert syndrome
1 patient with microhematuria
1 patient that referred history of renal lithiasis
1 patient with long-standing liver transplant, referred for periodic follow-up
1 patient with 2 focal nodular hyperplasia for annual follow-up
1 patient referred to characterize hypodense lesions in left hepatic lobe seen in CT
Requests for Thyroid Gland Ultrasound
1 patient with adenomatous familial polyposis syndrome referred to rule out papillary thyroid carcinoma
7 patients with nodular thyroid disease, including 4 multinodular goiters
2 patients with recent diagnosis of hypothyroidism
1 patient with clinical background of breast cancer and a hypermetabolic right thyroid nodule seen in PET-CT
1 patient with normocalcemic hyperparathyroidism

### Abdominal tele-robotic US learning curve

**Fig. 2:**
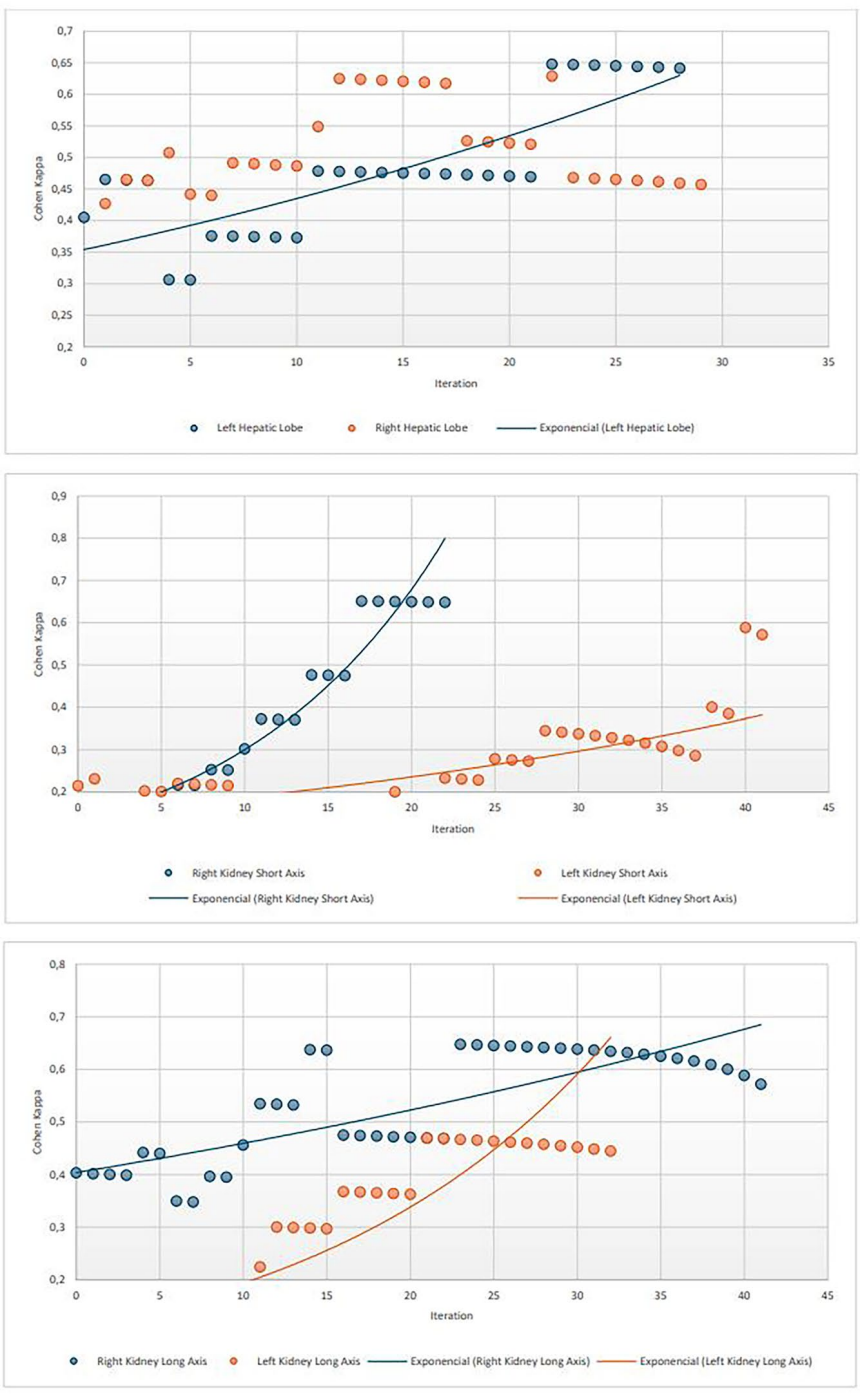
Evolution of the Kappa of Cohen and exponential relation through each iteration for the assessment of left and right hepatic liver (top graphic), right and left kidney short axis (mid graphic) and right and left kidney long axis (bottom graphic) when using the telerobotic US system. The total number of iterations was halved due to having null values (low variance) or perfect correlation in the last iterations

The evolution of Cohen’s kappa between tele-robotic US and standard US for left and right hepatic lobe and short and long axis of both kidneys in the 51 abdominal studies is depicted in Fig. 2.

The distribution of the Cohen’s kappa over the successive studies showed an exponential tendency with moderately high R^2^: 0.69 (for left hepatic lobe), 0.58 (for right kidney long axis), 0.92 (for right kidney short axis), 0.71 (for left kidney long axis) and 0.64 (for left kidney short axis). The right hepatic lobe did not show an exponential tendency, with R^2^ < 0.1. Moreover, the ANOVA test showed no significant differences (p > 0.05) between the predicted and real values of the variables following an exponential tendency.

Cohen’s kappa achieved moderate/substantial correlation with values above 0.6 for left hepatic lobe from the participant 22 onwards, being the highest one 0.65. For the long and short axis of the right kidney it achieved values above 0.6 from the participant 23 and 16 respectively, and the highest values achieved were approximately 0.65 in both variables. For the long axis of the left kidney, it achieved values above 0.40 from patient 21, being the highest one 0.45. Finally, for the short axis of the left kidney, it achieved values above 0.55 from the patient 40, being 0.58 the highest value.

The learning curve for the thyroid gland was not assessed since there was perfect correlation between the variables assessed in the standard and tele-robotic US, as shown later.

### Tele-robotic US time of exploration

**Fig. 3:**
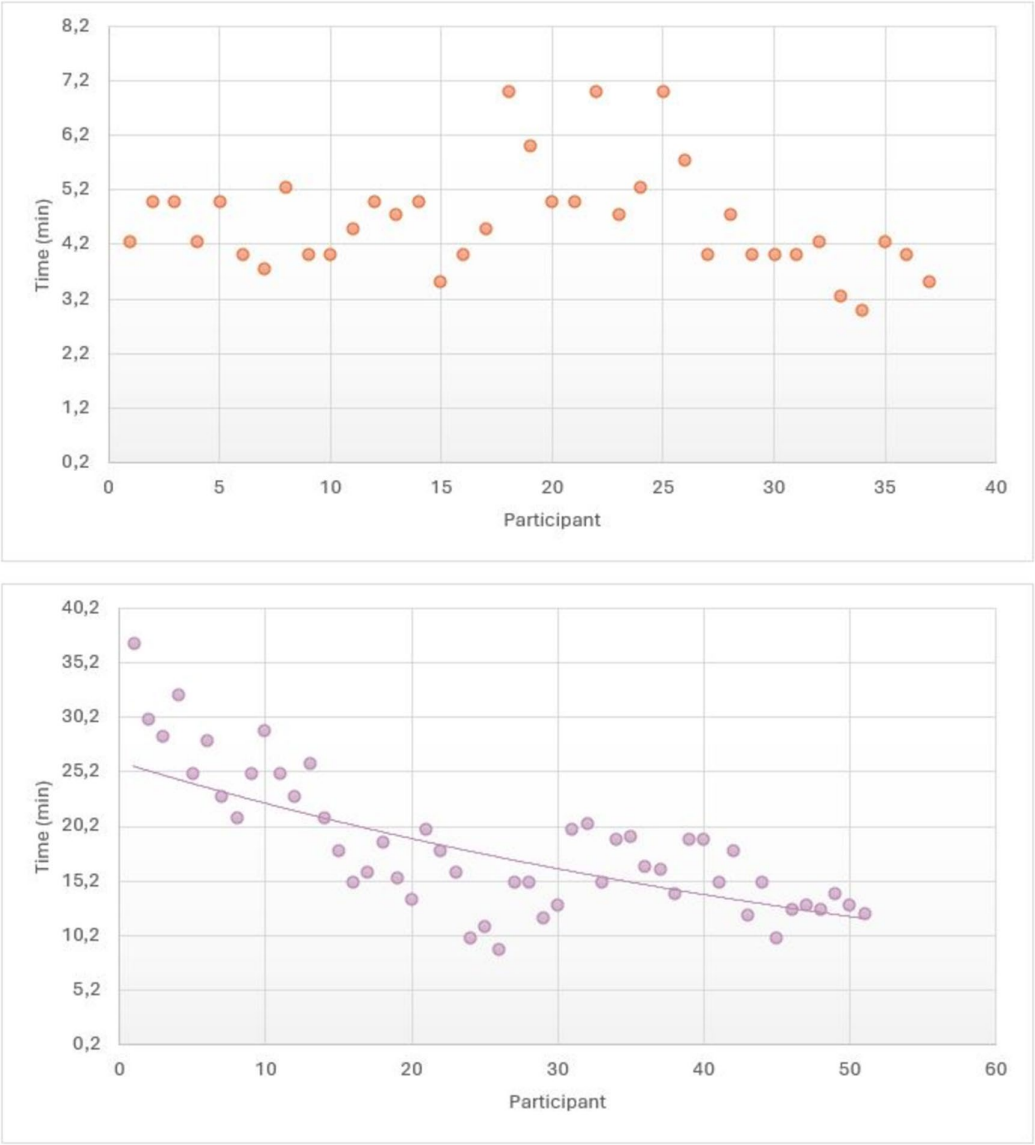
Evolution of the time required for performing the thyroid gland (top graphic) and abdominal (bottom graphic) explorations through the study, with the exponential relation in the case of the abdominal explorations

The mean time of exploration of the tele-robotic US for abdomen and thyroid gland examinations was 18.33 ± 6.26 min and 4.64 ± 0.97 min respectively. The evolution during the whole study is depicted in Fig. 3. The abdominal explorations showed a negative exponential growth (R^2^ 0.5), achieving a “plateau” from the 15–20th exploration, ranging between 10 and 20 min. The thyroid gland explorations showed no clear relation in time, without a perceptible decrease in the time required to perform the tele-robotic US.

### Comparison between tele-robotic and standard US

Based on the learning curve and the evolution of the time required to perform the tele-robotic abdominal US, an estimated cut-off value of 20 abdominal explorations was selected as the minimum to achieve a sufficient training to properly manage the robotic device. The minimum number for the thyroid gland exploration was not considered since the correlation was excellent from the beginning.

Barnard test’s results for the different variables recorded in the abdominal explorations are depicted in Table 3, including the results of the full cohort, pre-trained and post-trained cohort between tele-robotic and standard US.

**Table 3:** Results of the Barnard Test for the different qualitative variables assessed in the abdominal explorations

	Main Cohort (n = 51)		p value	Pre-trained Cohort (n = 20)		p value	Post-trained Cohort (n = 31)		p value
	Tele-robotic US	Standard US		Tele-robotic US	Standard US		Tele-robotic US	Standard US	
Left hepatic lobe	44 (86.3)	49 (96.1)	0.09	16 (80)	19 (95)	0.23	28 (90.3)	30 (96.8)	0.36
Right hepatic lobe	38 (74.5)	47 (92.2)	0.03	12 (60)	18 (90)	0.03	26 (83.4)	29 (93.5)	0.53
Hepatic veins	41 (80.4)	49 (96.1)	0.07	14 (70)	18 (90)	0.14	27 (87.1)	30 (96.8)	0.36
Portal vein	48 (94.1)	51 (100)	0.21	17 (85)	20 (100)	0.20	31 (100)	31 (100)	1
Gallbladder*	48 (96)	50 (100)	0.21	17 (89.5)	19 (100)	0.20	31 (100)	31 (100)	1
Pancreatic head	36 (70.6)	43 (84.3)	0.07	14 (70)	16 (80)	0.29	22 (71)	27 (87.1)	0.14
Right kidney short axis	40 (78.4)	50 (98)	< 0.01	11 (55)	20 (100)	< 0.01	27 (87.1)	30 (96.8)	0.68
Right kidney long axis	41 (80.4)	48 (94.1)	0.04	13 (65)	19 (95)	0.07	28 (90.3)	29 (93.5)	0.36
Left kidney short axis	32 (62.7)	48 (94.1)	< 0.01	9 (45)	18 (90)	< 0.01	23 (74.2)	30 (96.8)	0.03
Left kidney long axis	37 (72.5)	49 (96.1)	< 0.01	10 (50)	19 (95)	< 0.01	27 (87.1)	30 (96.8)	0.36
Urinary bladder**	45 (93.8)	48 (100)	0.09	17 (94.4)	18 (100)	0.20	28 (93.3)	30 (100)	0.52
Abdominal aorta	38 (74.5)	44 (86.3)	0.24	15 (75)	17 (85)	0.53	23 (74.2)	27 (87.1)	0.33

Data is expressed as total of positive observations, with percentage in parenthesis

^*^There was one patient with cholecistectomy so the n is 50 for the main cohort and 19 for the pre-trained cohort

^**^3 participants had a non-distended urinary bladder, so the n is 48 for the main cohort, 18 for the pre-trained cohort and 30 for the post-trained cohort

Right hepatic lobe and short/long axis of both kidneys show statistical differences (p < 0.05) between both techniques in the full cohort. These variables also showed statistically differences in the pre-trained cohort (p < 0.05) except the assessment of the right kidney in the long axis (p = 0.07), which showed no differences between both techniques. When trained, all variables showed no statistical differences (p > 0.05) except the visualization of the left kidney in the short axis (p = 0.03), which continued to be different between both techniques. The relative frequency of this variable in the pre-trained cohort is 45% and increases to 74.2% in the post-trained cohort.

The assessment of the left kidney in short axis was the variable less frequently assessable (62.7%) with the tele-robotic US in the full cohort, while the gallbladder was the most assessable (96%).

The Barnard test’s results for the different variables annotated in the thyroid gland explorations (long and short axis of left and right lobe, isthmus, left and right main cervical vessels and left and right submandibular glands) are not depicted since there was a perfect correlation between both techniques (p = 1).

The results for the paired samples t-test of the quantitative variables assessed in the last 31 patients are depicted in Table 4. The different variables assessed for abdominal and thyroid gland explorations showed no statistical differences (p > 0.05) between the measurements obtained through the tele-robotic and standard US. Only the long axis length of the left kidney was statistically different (p = 0.004) between both techniques, with a value of 11.24 ± 0.8 cm in the tele-robotic US group and 11.47 ± 0.82 cm in the standard US group.

**Table 4:** Assessment of the quantitative variables in the last 31 patients for abdominal and thyroid gland studies between both techniques

	Tele-robotic US	Standard US	p value
Abdominal exploration			
Portal vein velocity (cm/s)	22.28 ± 6.39	23.67 ± 5.23	0.1
Portal vein diameter (mm)	11.53 ± 1.83	11.1 ± 1.87	0.11
Right kidney long axis (cm)	11.16 ± 1.08	11.31 ± 0.93	0.22
Left kidney long axis (cm)	11.24 ± 0.8	11.47 ± 0.82	0.004
Spleen (cm)	10.49 ± 1.52	10.68 ± 1.28	0.14
Thyroid gland exploration			
Right lobe short axis (cm)	1.79 ± 0.48	1.83 ± 0.52	0.27
Right lobe long axis (cm)	4.08 ± 0.67	4.3 ± 0.81	0.05
Left lobe short axis (cm)	1.73 ± 0.52	1.79 ± 0.58	0.1
Left lobe long axis (cm)	4.17 ± 0.78	4.1 ± 0.73	0.41
Isthmus (mm)	3.02 ± 1.42	2.85 ± 1.36	0.23

Data is expressed as mean ± standard deviation

### US findings of tele-robotic examinations

22 incidental findings were found in the abdominal explorations performed with the tele-robotic US, whereas 25 were found using the standard US machine. The findings missed by the tele-robotic US were one small hepatic cyst of 9 mm in the right hepatic lobe and two left renal simple cysts in two patients in which the left kidney was not fully viewed with the tele-robotic US. Findings are depicted in Table 5.

**Table 5:** Abdominal incidental findings and thyroid nodules diagnosed with tele-robotic and standard US, detailing the specific finding and Thyroid Imaging Reporting and Data System (TI-RADS) category of the thyroid nodules

Abdominal incidental findings	Telerobotic US	Standard US
Liver steatosis	4	4
Hepatic cysts	5	6
Hepatic hemangiomas	2	2
Gallstone	2	2
Right renal cysts	5	5
Left renal cysts	3	5
Right hydronephrosis	1	1

Thyroid nodules	Telerobotic US	Standard US
Diffuse multinodular goiter	6	6
TI-RADS 2 nodules	12	12
TI-RADS 3 nodules	8	9
TI-RADS 4 nodules	1	1

In the thyroid gland explorations, all the thyroid nodules were identified with the tele-robotic examination except one small TI-RADS 2 nodule, described in Table 5. Spongiform, cystic nodules and small nodules of < 10 mm in multinodular goiter were excluded from the table due to its low relevance. There was one nodule classified as a TI-RADS 2 with the tele-robotic US but as a TI-RADS 3 with the standard US. This discrepancy is explained because there were nodular calcifications missed by the tele-robotic US.

### Patients’ assessment

The results of the different items assessed in the participant survey are presented in Table 6. Most participants (> 70%) felt comfortable and safe while being examined by tele-robotic US for abdominal and thyroid gland explorations, as well as a high satisfaction rate (> 80%) with the communication with the radiologist (Likert-scale value of 5).

**Table 6:** Results of the different questions assessed in the participant survey in the abdominal and thyroid gland explorations, in which each item is evaluated with a 5-point Likert scale

Abdominal explorations	Punctuation				
Item	1	2	3	4	5
Q1: I felt comfortable when being examined by the tele-robotic US	0 (0)	0 (0)	3 (5.9)	9 (17.6)	39 (76.5)
Q2: I felt safe when being examined by the tele-robotic US	0 (0)	0 (0)	3 (5.9)	9 (17.6)	39 (76.5)
Q3: The communication with the radiologist was fluent when being examined by the tele-robotic US	0 (0)	1 (2)	1 (2)	5 (10)	44 (86)
Q4: I got my questions about the procedure answered before being examined by the tele-robotic US	0 (0)	0 (0)	0 (0)	5 (9.8)	46 (90.2)
Q5: I trust the results obtained with the tele-robotic US	1 (2)	0 (0)	5 (9.8)	16 (31.3)	29 (56.9)
Q6: The tele-robotic US is more uncomfortable than the standard US	23 (45.1)	8 (15.7)	7 (13.7)	5 (9.8)	8 (15.7)
Q7: I felt a higher pressure in the examined zone with the tele-robotic US than with the standard US	36 (70.6)	4 (7.8)	3 (5.9)	7 (13.7)	1 (2)
Q8: I prefer the tele-robotic US rather than the standard US	4 (7.8)	14 (27.5)	25 (49)	2 (3.9)	6 (11.8)
Q9: I would be comfortable with the implementation of the tele-robotic US in the future	1 (2)	9 (17.6)	13 (25.5)	8 (15.7)	20 (39.2)

Thyroid gland explorations	Punctuation				
Item	1	2	3	4	5
Q1: I felt comfortable when being examined by the tele-robotic US	0 (0)	2 (5.4)	4 (10.8)	5 (13.5)	26 (70.3)
Q2: I felt safe when being examined by the tele-robotic US	0 (0)	2 (5.4)	2 (5.4)	5 (13.5)	28 (75.7)
Q3: The communication with the radiologist was fluent when being examined by the tele-robotic US	0 (0)	0 (0)	2 (5.4)	4 (10.8)	31 (83.8)
Q4: I got my questions about the procedure answered before being examined by the tele-robotic US	0 (0)	0 (0)	0 (0)	4 (10.8)	33 (89.2)
Q5: I trust the results obtained with the tele-robotic US	0 (0)	1 (2.7)	6 (16.2)	12 (32.4)	18 (48.7)
Q6: The tele-robotic US is more uncomfortable than the standard US	7 (19)	6 (16.2)	9 (24.3)	9 (24.3)	6 (16.2)
Q7: I felt a higher pressure in the examined zone with the tele-robotic US than with the standard US	8 (21.6)	2 (5.4)	7 (18.9)	11 (29.8)	9 (24.3)
Q8: I prefer the tele-robotic US rather than the standard US	2 (5.4)	9 (24.3)	24 (64.9)	1 (2.7)	1 (2.7)
Q9: I would be comfortable with the implementation of the tele-robotic US in the future	0 (0)	2 (5.4)	9 (24.3)	8 (21.6)	18 (48.7)

Numbers are expressed in absolute frequency and percentage in parenthesis

56.9% and 48.7% of the participants in the respective abdominal and thyroid gland cohort denoted a high level of confidence in the results of the tele-robotic US for diagnostic purposes (Likert-scale value of 5). However, most of the participants, 49% and 64.9% respectively, are indecisive (Likert-scale value of 3) whether they prefer tele-robotic US rather than standard US despite being mostly comfortable with its implementation in the future (39.2% and 48.7%, Likert-scale value of 5).

The main discrepancies are found when asked about the pressure exerted by the robotic arm. 70.6% of participants in the abdominal exploration cohort did not feel higher pressure than the one experienced with the standard US (Likert-scale value of 1). This contrasts with what participants reported for the thyroid gland examination, in which there is a broader spectrum of sensations but there was a tendency to feel higher pressure than the one felt with the standard US.

## Discussion

The first preclinical tele-robotic models were first developed in the late 90’s and beginning of the twenty-first century, which were aimed at different clinical applications [11–13].

One of the first models to be commercially available for clinical applications was the MELODY system in France. It was the first model to use a mock US probe with a 3-DOF manipulator but required an assistant at the patient site to perform the compression and sliding of the probe [14, 15].

Since then, different models have been commercialized for clinical use, improving the overall design such as a higher DOF (up to 6–7) to reduce the intervention of the assistant. Other improvements included diminishing the latency between the doctor’s and patient’s end as well as better audiovisual communication and real-time US parameters adjustment. The history, development, and future directions of the tele-robotic US are beyond the scope of this article. We refer to two published reviews that deepen in this manner [5, 16].

Since the commercialization of the first clinical models there have been several studies assessing the feasibility of the tele-robotic US, generally using the standard US as a reference. There have been studies conducted in abdominal, obstetric, trauma/musculoskeletal, echocardiography, vascular and even thyroid imaging [8, 9, 14, 17–25].

The first published studies in abdominal imaging were done with the MELODY system and its precursors. Several studies in the first decade of the twenty-first century assessed different abdominal organs and structures such as the liver, portal vein, gallbladder, pancreas, spleen, kidneys, bladder, aorta and urogenital structures [17–20].

The visualization rate of the reported structures was generally above 80%, although some studies reported a lower visualization rate in some organs such as the spleen [17] and pancreas [18].

In 2017, Adams et al. evaluated the MELODY system in abdominal imaging in a cohort of 18 patients. 92% of the abdominal organs were correctly visualized with the tele-robotic US and it was also one of the first to assess quantitative measurements such as kidneys sagittal length and spleen long axis among others. There were no statistically significant differences when measuring liver parameters, spleen and proximal aorta but showed differences when assessing both kidneys in sagittal length, common bile duct diameter and distal aorta diameter [14].

Later, Adams et al. (2022) also conducted another study with the MELODY system in a remote area with 82 patients and a total of 87 explorations, of which 35 were abdominal. Among all the studies, 28% were considered inadequate for diagnosis and the rest adequate or adequate with reservations [21].

The era of 5G technology has allowed the development of new clinical studies with improved tele-robotic devices. Duan et al. (2021) evaluated the use of tele-robotic US in in the Intensive Care Unit in a total of 33 patients and assessed several diagnostic and incidental findings in the abdominal organs including the liver, gallbladder, pancreas, spleen, kidney as well as examinations for pleural and abdominal effusion. There was a moderate consistency between both techniques with a Kappa value of 0.6 [9]. Zhang et al. (2024), found no significant differences when measuring the aorta, portal vein, gallbladder, and kidney (longitudinal diameter). However, tele-robotic US underestimated the transverse diameter of the kidney [22].

Our results are consistent with the reported literature when assessing abdominal structures. There was an overall moderate/substantial correlation when assessing the left hepatic lobe and short and long axis of both kidneys in the post-trained cohort. There were also no statistical differences when assessing all the quantitative and qualitative variables in the post-trained cohort except the visual assessment of short axis of the left kidney and the measurement of the long axis of such kidney. In the latter, the difference between both means was approximately 2 mm with similar standard deviation, so it is not clinically relevant. Moreover, most of the incidental findings were also captured with the tele-robotic system including liver steatosis, gallstones or hydronephrosis. A few small hepatic and renal simple cysts were missed by the tele-robotic US. This is in line with other published studies [23–25].

Based on the results and the differences between the pre-trained and post-trained cohorts, the liver (especially the right lobe) and kidneys are the hardest structures to evaluate with the tele-robotic US. The location of these structures demands the reposition of the transducer in specific angles or planes in comparison to other structures such as the aorta, pancreas or urinary bladder that are easily accessible in supine position in a standard plane. The limited movement of the robotic arm in comparison to the hand, although being excellent, can contribute to these organs being the hardest to evaluate. Also, the lack of left-side video-camera limits the visualization of left-side structures like the kidneys. In such cases, the portable video-camera was allocated at the left side of the patient.

Another important factor to be considered is the presence of a marked subcostal liver, which limits the assessment of this organ through the subcostal plane. In these cases, the liver is better assessed through the intercostal plane but, in our experience, it is hard to master the visualization of this organ through this plane with the tele-robotic US. We hypothesize that a limited visualization and perspective of the ribs may contribute to this limitation.

Some other limitations found when evaluating these structures were increased body habitus, abundant bowel gas or presence of Chilaiditi syndrome, as also noted in other studies [25].

It is yet to be seen if more experience and training is required to halve these limitations in evaluating these structures.

Patient collaboration is also paramount for a good assessment since participants were frequently asked to move into a specific position or take a deep breath to better assess some structures such as the kidneys. As noted, restrictions in arm movement that complicates reaching some locations might contribute to this dependency. A study made with patients with COVID-19 pneumonia noted that the poor cooperation of the patients was one of the factors that difficulted performing the tele-robotic US [23].

We did not evaluate the use of tele-robotic US in patients seeking urgent medical attention. The fact that some structures such as the gallbladder and the kidneys were correctly assessed with this technique could highlight its role emergency radiology, especially in remote areas or patients that need to be isolated. Duan et al. (2021) found good correlation in a cohort of patients in the Intensive Care Unit when assessing gallbladder wall thickening, enlarged gallbladder, cholecystitis or bilateral hydronephrosis. Only two cases of hepatic cysts and one gallbladder polyp were missed by the telerobotic US, which per se are not relevant findings in patients with acute abdomen [9].

The caliber of the common bile duct was not assessed in our study because it was not considered to be feasible in non-emergency patients. Moreover, the higher resolution of the standard US might allow the visualization of the non-dilated common bile duct whereas this will not be achieved with the tele-robotic US and produce a bias. However, since the portal vein was assessable in almost all cases, it is presumably possible to detect a dilated common bile duct in the portal space.

Further studies are needed to better assess the role of tele-robotic US in patients admitted into the emergency room, as well as other clinical scenarios. Depending on the intended use, future tele-robotic US should be coupled with technically better US models.

The evaluation of the thyroid gland showed excellent concordance with the standard US, as shown in results. Zhang et al. (2022) did a comprehensive assessment of the clinical use of 5G-based tele-robotic US in thyroid disease in comparison to conventional US in a cohort of 139 patients. As in our case, there were not statistically significant differences between the measurements of the thyroid gland [8].

We also assessed thyroid nodules based on TI-RADS classification. All the nodules were correctly assessed except one TI-RADS 2 nodule which was missed by the tele-robotic US. Nodules were also correctly classified with the tele-robotic US except one TI-RADS 3 nodule that was misclassified as a TI-RADS 2. The tele-robotic US failed to detect calcifications in the cystic-solid nodule. Our results are in concordance with what Zhang et al. (2022) reported, in which only five of more than 100 nodules were missed by the tele-robotic US, and all of them were TI-RADS 3 [8].

The quasi-flat surface of the neck with easily accessible superficial structures allows for a good examination with the tele-robotic US. Lack of patient collaboration besides neck hyperextension and absence of conditions that may disturb the examination such as intestinal gas also facilitates the use of this technique. Moreover, thyroid disease is highly prevalent, and US is the gold-standard imaging modality for its assessment. In the light of the above, we believe thyroid examinations might be the first ones to be habitually performed by tele-robotic US, especially in remote areas.

To the best of our knowledge, this is the first study that an estimated learning curve for this technique is given. Based on our results the learning curve for achieving a good performance with tele-robotic US is fast and assessable. There was a decrease in the time needed to perform the US specially during the first 20 studies, and better overall results after that mark in abdominal explorations. In thyroid gland explorations there was an even faster learning curve, being almost excellent since the first patients.

We did not assess the learning curve in more than one radiologist. Ren et al. evaluated the variation trend of examination time in two sonographers and noted a downward trend despite the fluctuations for both. These results emphasize the progressive evaluation time reduction due improvement of experience and proficiency with the tele-robotic US [25].

The average time for performing the abdominal and thyroid gland explorations is in line with other studies that use a similar device, although there is a clear heterogeneity in the reported literature, probably because of differences in the protocol [9, 25].

Although not assessed in this study, the examination time is longer in tele-robotic US compared to standard US as demonstrated in the published literature [14, 22].

There was a high satisfaction/agreement rate when assessing the safeness and comfort of the tele-robotic US and communication with the radiologist. This is in line with other reported studies [8, 14, 21, 25].

Some patients reported excessive neck pressure during thyroid examination, a finding that has been previously described. Zhang et al. (2022) hypothesized that this may be due to the lack of adequate subcutaneous fat to reduce the force of US transducer and the cylindrical shape of the neck, which makes it harder for the probe to fit [8]. We believe the pressure on the neck should be also reduced to avoid this discomfort.

There was also indecisiveness about which technique is preferred. We believe that it will gradually gain more acceptance once it is implemented, as the performance is on par with the standard US. The possibility of reducing patient travel time might contribute to its acceptance, as noted in other studies [21].

A favorable cost–benefit is also important for the clinical implementation of tele-robotic US. There are few cost-analysis studies in which the feasibility of tele-robotic US has been assessed. Löfgren et al. found no clear cost differences at healthcare administration level if distant tele-robotic US was used instead of on-site traditional US for heart failure assessment with echocardiography. However, there was a reduced cost at personal level due to the patient-related costs (such as traveling) [26]. In a cost-minimization study, Adams et al. found that tele-robotic US and/or itinerant sonographer had a lower average cost than traveling radiologist/sonographer in rural and remote communities at healthcare and personal perspective [27]. Other studies have also shown affordable costs of this technique in rural areas [28]. More studies are needed to determine if tele-robotic US is economically viable in different scenarios besides rural and remote communities.

Patients, clinicians and healthcare providers acceptance are therefore paramount for the implementation of this technique in clinical scenarios, as well as appropriate regulatory policies [16]. Future studies will also determine the best scenario for tele-robotic US, whether it is in areas with low access to medical resources, emergency departments or other situations. Finally, the inception of artificial intelligence has the potential to strengthen the use of this technology. Autonomous robotic US might be able to scan several patients with minimal or without human intervention and detect relevant findings such as free fluid in polytraumatic patients. Also, it might be able to do standard examinations in outpatients which can be later tele-reviewed by a radiologist or specialist. This technology, however, is still under development [16].

There are several limitations in our study. First, the cohort in which the study was conducted had a normal mean BMI and patients were able to properly collaborate when performing the tele-robotic US, so the results may be overestimated for real-case scenarios. Future studies should aim at exploring the use of tele-robotic US in non-ideal scenarios such as patients with high BMI or impaired diseases. It is also especially important to assess its feasibility in patients seeking urgent medical attention in rural or remote communities due to the limited personnel resources in such places. Second, a single radiologist was responsible of conducting the tele-robotic US, not allowing for a comparison between different radiologists. Although relevant, the ability to correctly use the tele-robotic US also depends on the standard US experience of the subject. Third, the differences in the lower technical characteristics of the US model coupled with the robotic arm in comparison to the device used for the standard US can underestimate the tele-robotic results.

In conclusion, tele-robotic US has a good correlation with the standard US when evaluating abdominal structures, as well as a relatively fast initial learning curve and good overall acceptance. The performance when assessing the thyroid gland is even better, being almost identical to the standard US, which makes it a strong candidate for a future implementation. Despite these results, more clinical studies are needed to demonstrate its applicability in the daily clinical practice, especially in specific scenarios such as health checks-up or emergency departments.

## Supplementary Information

Additional file 1

## Data Availability

The datasets used and/or analyzed during the current study are available from the corresponding author on reasonable request.

## Abbreviations

US: Ultrasound
BMI: Body mass index
TI-RADS: Thyroid imaging, reporting and data system
DOF: Degrees of freedom
ANOVA: Analysis of variance
