# Supplementary material for: Validation of a tele-robotic ultrasound system for abdomen and thyroid gland explorations: a comparison with standard ultrasound
Source: Ultrasound J. 2025 Jan 13;17:2. doi: 10.1186/s13089-025-00408-6 (PMC11729597; doi:10.1186/s13089-025-00408-6)
Supplement: Supplementary file 1 — Additional file 1 [file 13089_2025_408_MOESM1_ESM.docx]

**SUPPLEMENTARY MATERIAL**

**TABLES S1-S2**

**Table S1.** Description of the qualitative and quantitative variables assessed in the thyroid gland explorations.

| **THYROID GLAND ULTRASOUND** | |
| --- | --- |
| **Qualitative Variables** | **Description** |
| **Right lobe short and long axis (yes/no)^a^** | Depiction of the right lobe in the short and long axis, from cranial to caudal and medial to lateral |
| **Left lobe short and long axis (yes/no)^a^** | Depiction of the left lobe in the short and long axis, from cranial to caudal and medial to lateral |
| **Isthmus (yes/no)** | Depiction of the thyroid isthmus in an axial plane |
| **Main left and right cervical vessels (yes/no)^a^** | Depiction of the carotid artery and internal jugular vein alongside its color Doppler signal at the level of the thyroid gland |
| **Left and right submandibular gland (yes/no)^a^** | Depiction of the submandibular gland |
| **Quantitative Variables** | **Description** |
| **Right lobe short and long axis (cm)^a^** | Measurement of the largest short axis and long axis in the most suitable plane for obtaining those values |
| **Left lobe short and long axis (cm)^a^** | Measurement of the largest short axis and long axis in the most suitable plane for obtaining those values |
| **Isthmus (mm)** | Measurement of the greater thickness of the isthmus in an axial plane |
| ^a^ Symmetric variables (such as left and right submandibular gland) or related variables (such as right lobe short and long axis) are included in the same cell for optimizing visualization but are considered two independent variables in the study. | |

**Table S2.** Description of the qualitative and quantitative variables assessed in the abdominal explorations.

| **ABDOMINAL ULTRASOUND** | |
| --- | --- |
| **Qualitative Variables** | **Description** |
| **Left hepatic lobe (yes/no)** | Depiction of the left hepatic lobe in different planes, until most of it can be assessed |
| **Right hepatic lobe (yes/no)** | Depiction of the right hepatic lobe in different planes, until most of it can be assessed |
| **Portal vein (yes/no)** | Depiction of the main portal vein in the hepatic hilum or proximal left or right hepatic portal vein. If it is partially visualized with B mode but color Doppler signal is well depicted, then it is considered as a “yes” |
| **Gallbladder (yes/no)** | Depiction of the whole gallbladder |
| **Pancreatic head (yes/no)** | Depiction of pancreatic head in an axial plane in the epigastric region. Due to the limitation of this technique in evaluating the pancreas a partial visualization of the pancreatic head is regarded as a “yes” |
| **Right kidney short and long axis (yes/no)^a^** | Depiction of the right kidney in long and short axis, from cranial to caudal and medial to lateral |
| **Left kidney short and long axis (yes/no)^a^** | Depiction of the left kidney in long and short axis, from cranial to caudal and medial to lateral |
| **Urinary bladder (yes/no)** | Depiction of the urinary bladder in axial and sagittal axis. Partial visualization of the bladder in the sagittal axis is acceptable due to the acoustic shadow of pubic symphysis |
| **Abdominal aorta (yes/no)** | Depiction of abdominal aorta in B mode and Doppler color mode until iliac bifurcation. Partial visualization of some segments is acceptable due to intestinal gas |
| **Quantitative Variables** | **Description** |
| **Portal vein velocity (cm/s)** | Measurement of the portal vein velocity in main portal vein or proximal right portal vein, with an angle between 30º-60º either through the intercostal or subcostal window |
| **Portal vein diameter (mm)** | Measurement of the main portal vein diameter either through the intercostal or subcostal window |
| **Right and left kidney long axis length (cm)^a^** | Measurement of the greater long axis length of both kidneys, from the tip of the upper pole to tip of the lower pole. If a long axis view from pole to pole was not achieved, then this variable was not measured |
| **Spleen (cm)** | Measurement of the greater achievable long axis length of the spleen |
| ^a^ Symmetric variables (such as left and right kidney) are included in the same cell for optimizing visualization but are considered two independent variables in the study | |

**FIGURES S1-S5**


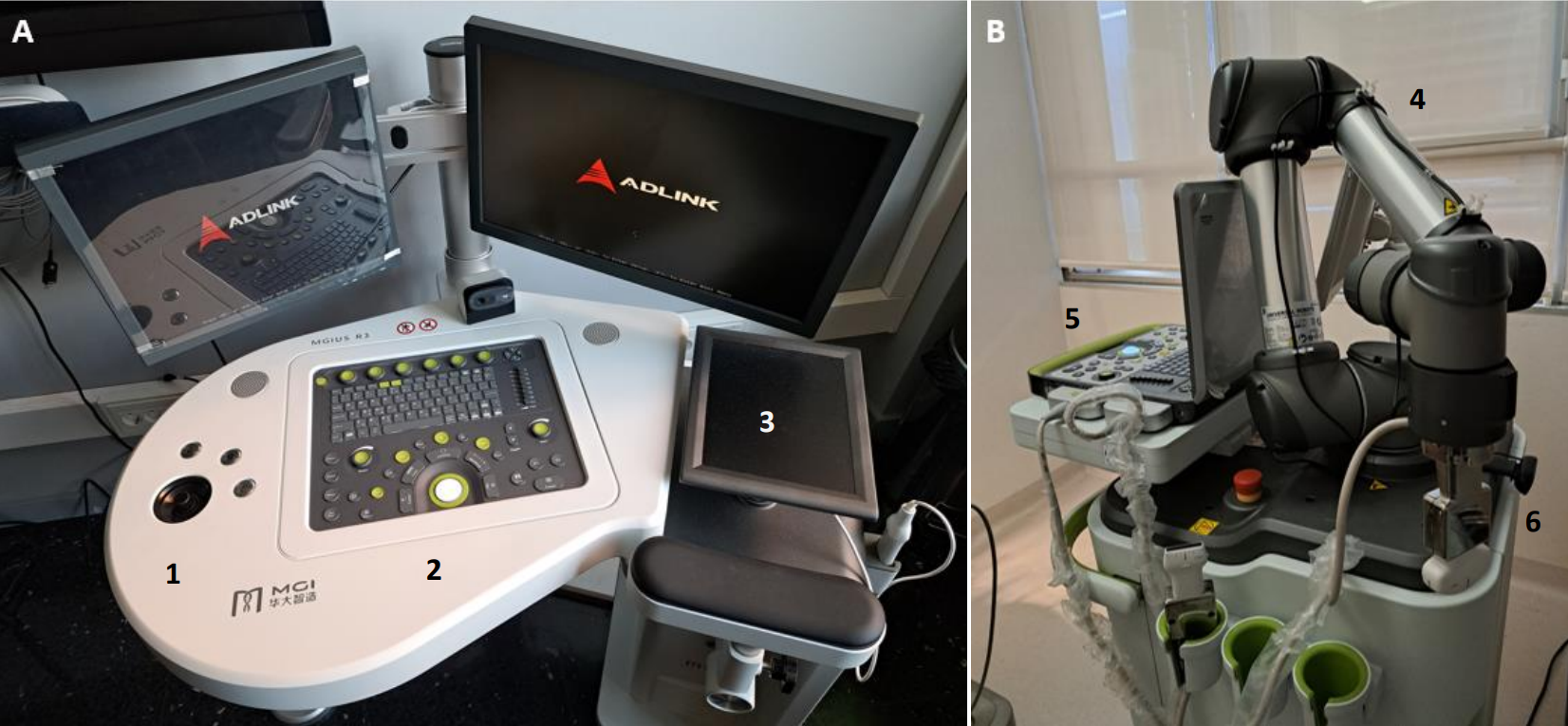


**Figure S1**. A depiction of the doctor’s end (A) and patient’s end (B), situated in different rooms. The doctor’s end includes the audiovisual control system (1), that allows switching between two cameras. One is stationary and located at the right-side of the patient and the other one is mobile (tripod system) and can be allocated anywhere, preferably at the patient’s feet and pointing towards them. The control system also allows the movement of the tripod camera. There is also the US control panel (2) and the robot-control console (3), which allows the remote movement of the robotic arm via a mock US probe and a simulation panel. The patient’s end contains, among other things, the robotic arm (4) that can accommodate the US transducer, as well as the US device (5) and the stationary right-sided camera (6, embedded in the portable structure).


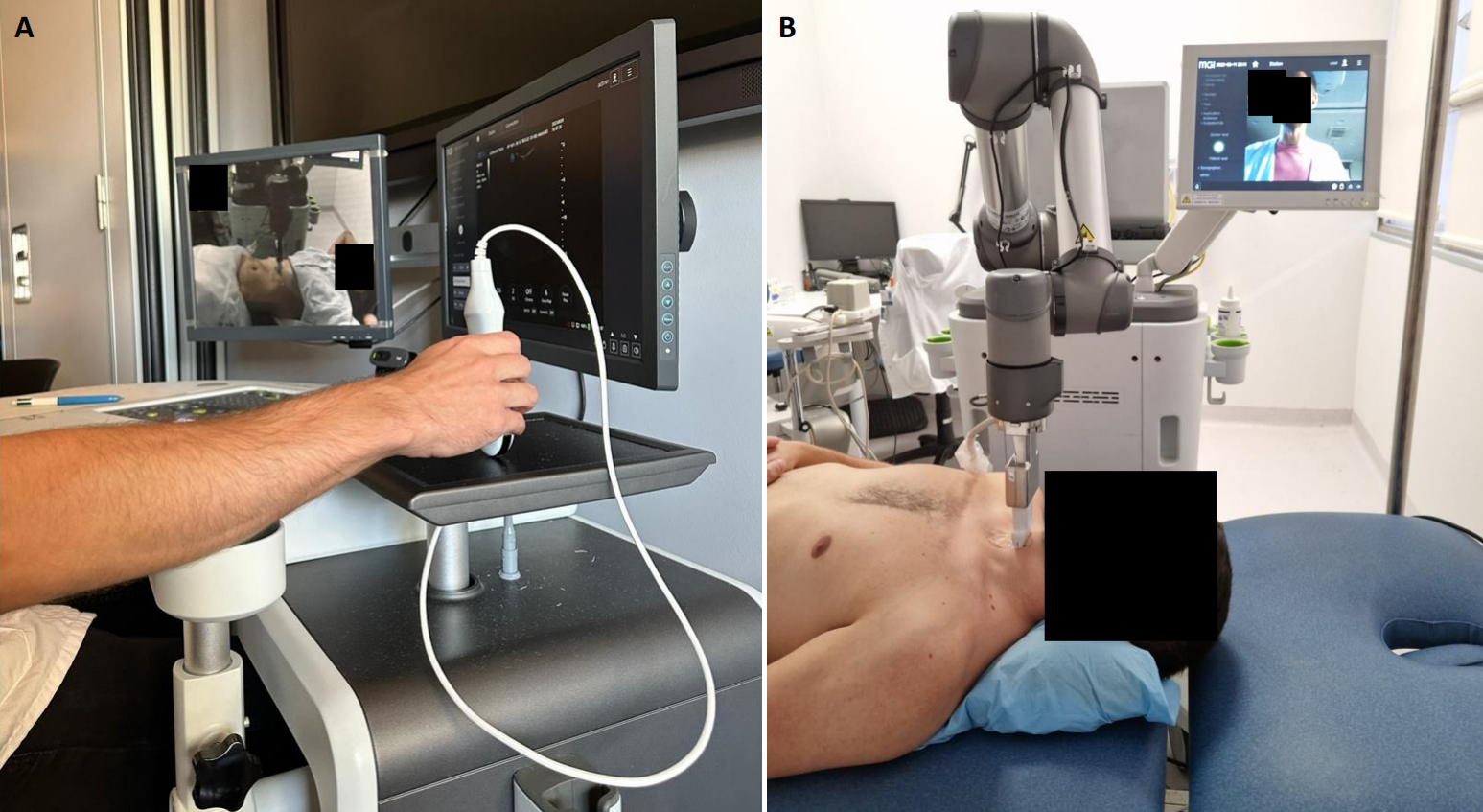


**Figure S2**. A depiction of the mock US probe held with the right hand and the simulation panel to control the robotic arm is depicted in A. In the left monitor the real-time image of the portable tripod-like video camera is shown, allowing a direct visualization of the US transducer attached to the robotic arm. In this case the camera was located at the patient’s left side. In the right monitor the real-time US images are displayed. The patient’s side view of a thyroid gland US is represented in B, with real-time audiovisual communication between the radiologist and the patient, as shown on the screen.


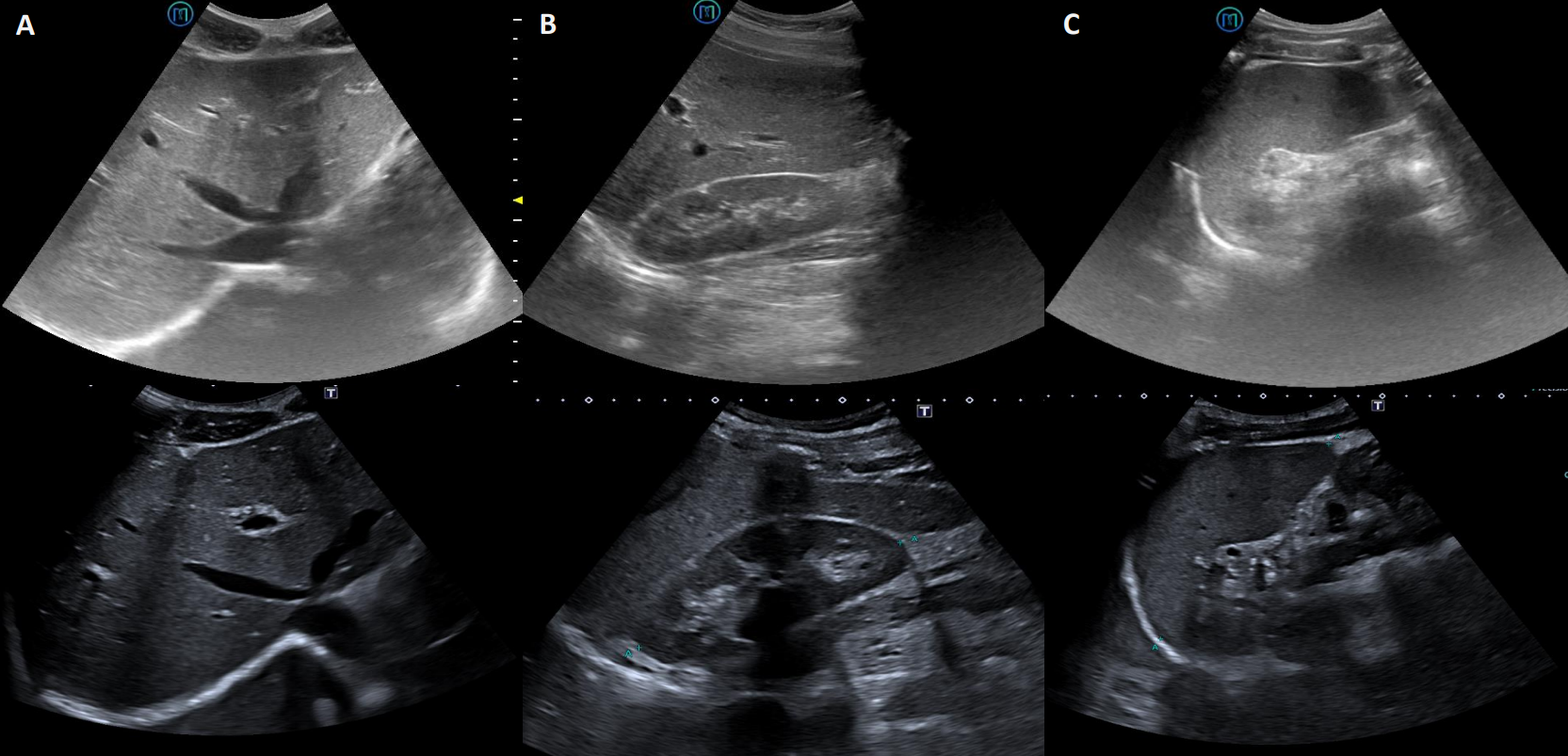


**Figure S3.** Comparison of abdominal US images obtained from the liver (A), right kidney (B) and spleen (C) between the tele-robotic US (upper row) and the standard US (lower row).


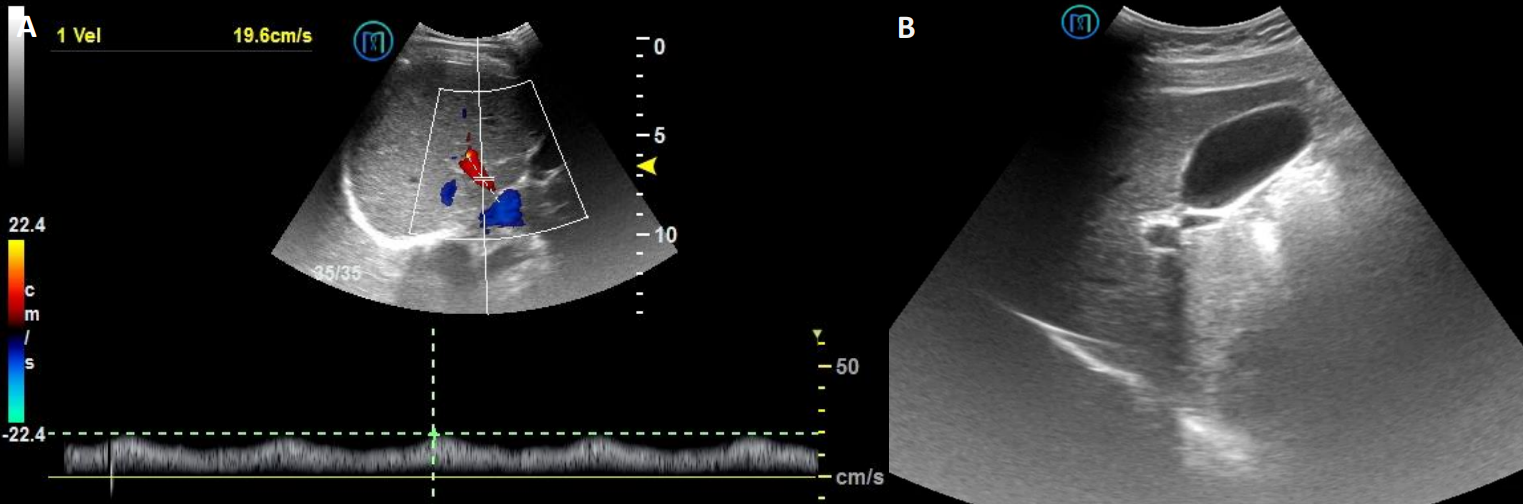


**Figure S4.** Tele-robotic US images obtained when measuring portal velocity (A) and depiction of the gallbladder (B).


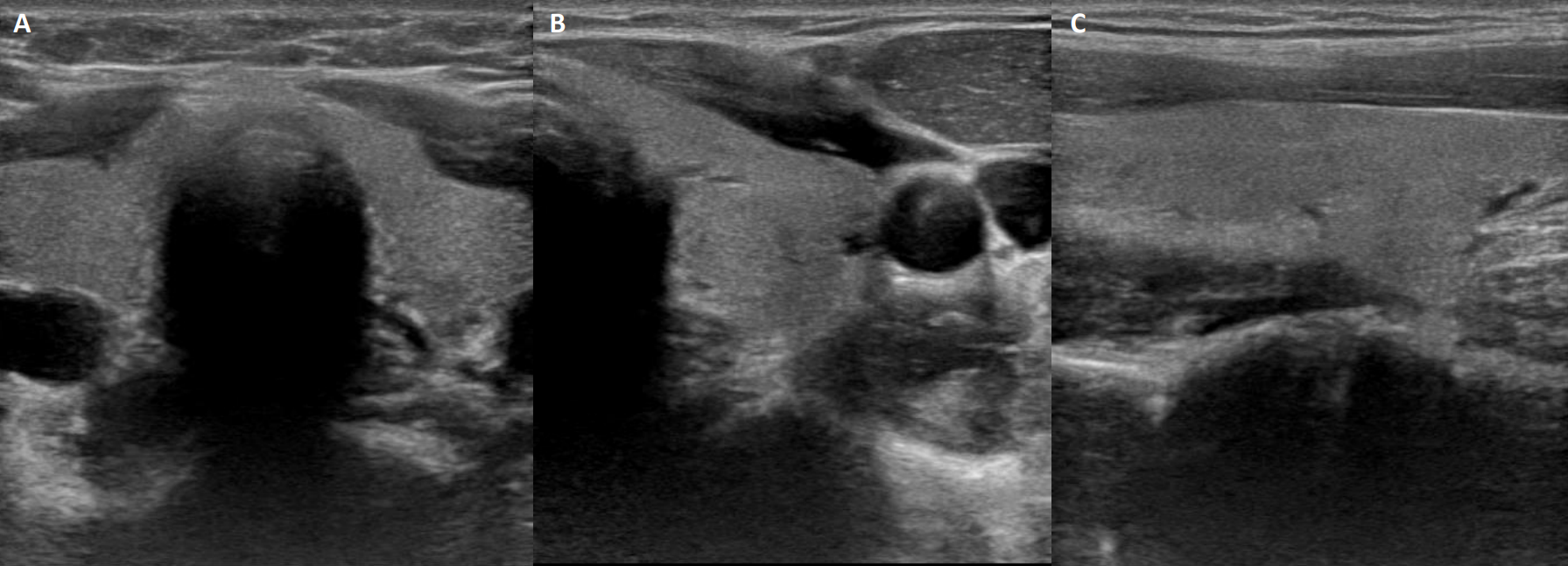


**Figure S5.** US images of the thyroid gland obtained through the tele-robotic US system. In A a full axial view of the thyroid gland is depicted. In B and C a short axis view (B) and long axis view (C) of the left thyroid lobe are shown.
